# Self-delivered misinformation - Merging the choice blindness and misinformation effect paradigms

**DOI:** 10.1371/journal.pone.0173606

**Published:** 2017-03-08

**Authors:** Lotta Stille, Emelie Norin, Sverker Sikström

**Affiliations:** Department of Psychology, Lund University, Lund, Sweden; Ludwig-Maximilians-Universitat Munchen, GERMANY

## Abstract

*Choice blindness* is the failure to detect a discrepancy between a choice and its outcome. *The misinformation effect* occurs when the recollection of an event changes because new, misleading information about the event is received. The purpose of this study was to merge the choice blindness and misinformation effect paradigms, and thus examine whether choice blindness can be created for individuals’ recollections of a witnessed event, and whether this will affect their later recollections of the event. Thus, as a way of delivering misinformation the participants ostensibly became their own source of the misleading information. The participants watched a short film and filled out a questionnaire about events shown in the film. Some of their answers were then manipulated using reattachable stickers, which allowed alteration of their original answers. The participants gave justifications for their manipulated choices, and later their recollection of the original event was tested through another questionnaire. Choice blindness was created for a majority of the participants. A majority of the choice blind participants later changed their reported recollection of the event in line with the manipulations, whereas only a small minority of the participants in the control condition changed their recollection. This study provides new information about the misinformation effect, suggesting that this effect also can occur when misinformation is given immediately following presentation of the original stimuli, and about choice blindness and its effects on the recollections of events. The results suggest that *memory blindness* can be created when people inadvertently supply themselves with misleading information about an event, causing a change in their recollection.

## Introduction

For the moment he had shut his ears to the remoter noises and was listening to the stuff that streamed out of the telescreen. It appeared that there had even been demonstrations to thank Big Brother for raising the chocolate ration to twenty grammes a week. And only yesterday, he reflected, it had been announced that the ration was to be REDUCED to twenty grammes a week. Was it possible that they could swallow that, after only twenty-four hours? Yes, they swallowed it. ([1], p. 32)

In the novel Nineteen Eighty-four, George Orwell describes the dystopian Oceania, where the past is constantly updated in order to correspond with the current stance of the Party. Winston Smith, a clerk in the Ministry of Truth, rewrites newspapers and other documents in accordance with the ever-changing statements made by Big Brother and the Party. He incinerates the old versions by throwing them down the memory hole. In this way the memories of the citizens are being imperceptibly manipulated. This sounds like an absurd procedure, but is it impossible to implement? Many would probably argue that they never could be tricked in such a way, but perhaps we overestimate our ability to detect when our memories do not correspond with the way things actually were, even when the manipulation occurs right in front of our eyes.

*Choice blindness* is the failure to detect a discrepancy between a choice and its outcome and to also justify this false outcome [2]. To create choice blindness, the choices made are manipulated so that the outcome of choice differs from the choice made by the participant. To illustrate, in the first published study on choice blindness [2] the researcher held up two pictures of female faces. By pointing, the participant indicated which of the two they considered the most attractive. After this, the researcher turned the pictures face down on the table and pushed the chosen face towards the participant. However, using a cunning card trick to switch the faces, the participant ended up with the picture they had in fact *not* chosen. Not only did the participants fail to detect this manipulation, but they could also justify why they had chosen the face they ended up with, even though they had initially preferred the other face. Choice blindness is a phenomenon that has been shown to span a wide variety of preferences: attractiveness [2], morality [3], politics [4] and finance [5]. It has been created for different senses: smells and tastes [6], tactility [7], and auditive choices [8, 9].

Standard procedure in choice blindness studies is to conclude each experiment by asking a number of detection questions to establish whether the participants at any point suspected manipulation of their answers. The questions are gradually made more explicit, e.g. “If one of your answers had been altered do you think you would have noticed?” This is done to make sure that the phenomenon is not based on social desirability and that participants do not simply try to comply with the experimenters. If no such reporting occurs, this is labeled “choice blindness blindness”, that is, the participant has been blind to their own blindness [2, 10].

The choices that participants believe they have made can be stable over time. Hence, the manipulations can create prolonged preference changes and affect future choices [11, 12]. However, little research has focused on the memory aspect of choice blindness.

In a field study, Sagana et al. [13] explored whether choice blindness could be created for eyewitnesses’ recognition of faces. From a photo line-up, participants picked out two pretend tourists they had briefly spoken to. One of their choices was then manipulated, that is, when confronted with their choices, participants were told they had picked a different photo from the one they had actually picked and were asked to justify this choice. Choice blindness was created for 41% of the manipulated items. Sagana et al. [10] followed this up by further investigating recognition of faces. In a number of experiments, the participants first witnessed mock-crimes on film. From a photo line-up they then picked out people involved in the crimes and these choices were then manipulated. Choice blindness was either completely absent or created for a meager 6% of the participants. To investigate whether the manipulations would affect future choices, the participants came back 24 hours later to make the same choices once again. The manipulations did not affect these choices; only two out of 37 participants chose differently than they had in the original line-up, and none of these were consistent with the manipulations.

In two recent experiments, both conducted online, Cochran et al. [14] let participants view slides depicting criminal events, and then either report their memories for episodic details of the witnessed event or identify a suspect from a photo line-up. Later they were confronted with their own reported recollections and identifications respectively, but some of these had been manipulated in regular choice blindness fashion. Finally, the participants were once again asked to give their recollections of the criminal events, in order to test possible memory distortions.

The results of the first experiment, on episodic details, showed that for manipulated items, but not for control items, the responses appeared to change between the two testings. There were significant main effects for manipulated items as compared to control items, as well as for the test occasion, and a significant interaction between these two variables. However, as the authors themselves contend, a non-stringent way of measuring detection was used. They could therefore not conclude whether it was a created choice blindness or the mere exposure to a manipulation that gave this change in recollection between the two testings.

The second experiment, on identifications, extends the works of Sagana et al. [10, 13], which in turn, to a large extent, replicate previous studies of choice blindness showing that people fail to detect the manipulation of faces [2, 11, 15–17]. Whereas Sagana et al. were not able to create a long-term change in recollection, Cochran et al. [14] achieve a considerable misinformation effect (see below) as 54% of the non-detectors changed their pick of suspect and of these, 57% changed it in line with the manipulations. The authors interpret this as indication of a more long-term effect of choice blindness on the recollection of a line-up. Based on these results the authors introduce the term *memory blindness*–the effect of choice blindness on eyewitness recollection.

Choice blindness has also been examined for autobiographical memory [18]. Participants filled out a questionnaire concerning their own history of norm violating behavior. Some of the answers were later manipulated to reflect a different answer than the one originally intended. In the different conditions choice blindness was created for a mere 10% of the manipulated items, the rest were detected. Hence, this study achieved a very small effect size for choice blindness for memories in one’s far past.

To summarize, to our knowledge, no previous study has managed to show a clear connection between choice blindness for the retelling of a recently witnessed event and its potential effect on future recollections of that event.

The *misinformation effect* occurs when the recollection of an event changes because new, faulty information about the event is received [19], and the effect has been replicated in hundreds of studies [20]. The experimental paradigm used to test the misinformation effect usually contains three steps. First, the participants witness an event, ordinarily on film. After this, new false information is given about some course of events or details in the film. Finally, the participants are tested on their recollection of the original event to see if any changes have occurred [21].

The source of the misinformation is found to influence the creation of a misinformation effect [22]. A number of studies have found that the misinformation effect can occur after a discussion with a co-witness [23, 24] or after reading statements ostensibly written by another observer of the event [25]. The reliability of the source is shown to be important [22]. This was first studied in two experiments by letting participants first watch slides of a car accident [26]. They then answered questions or read narratives containing misinformation seemingly stemming from either an unbiased source (i.e. innocent bystander or unspecified) or a biased source (i.e. guilty driver or his lawyer). In a test of recollection where critical details were examined, the results indicated that the participants were more likely to be affected by the misinformation when they thought it came from an unbiased and reliable source. Further studies have followed with similar results. A larger misinformation effect has in a comparable way been achieved when the source was highly knowledgeable rather than completely naïve about the event [27]; had high-credibility rather than low-credibility as represented by a memory psychologist and a four-year old boy respectively [28]; had highly rated power and social attractiveness rather than low rated power and attractiveness [29]. Similar results of source reliability influence on a misinformation effect have also been found in studies concerning children’s recollections of witnessed events [30, 31].

Previous studies have encouraged participants to, in different ways, produce the misinformation themselves. This has been done either by forcing participants to confabulate [32, 33] or by encouraging them to generate incorrect information [34] about a witnessed event. Zaragoza and colleagues [32, 33] had the participants *knowingly* create confabulated memories and when later tested on their recollections they misattributed the source. In Roediger et al. [34] the false recollections were created through repeated retrieval of the misinformation the participants helped produce. However, the faulty information originally stemmed from a misleading narrative rather than originating from the participants themselves. In contrast, in the current study the participants are misled about their own past memory reports and though the misinformation is planted by the experimenters, from the participants’ viewpoint it derives completely from their own minds.

Other variables, besides the source, are found to influence a misinformation effect, for example an immediate retrieval of the event, what details are examined, and the time passage between the three experimental steps.

Studies of the impact of immediate retrieval of a witnessed event have generated conflicting results [35]. Some studies have demonstrated that such repeated testing in an eyewitness situation reduce susceptibility to misinformation and can even work as an immunizer against a misinformation effect [36–38]. This is in line with the testing effect, the phenomenon that a test of recollection enhances later retention [39]. By contrast, Chan and colleagues have in a number of studies demonstrated a reversed testing effect, i.e. that immediate retrieval of the witnessed event instead increases susceptibility to misinformation [40–42]. This reversed testing effect has been demonstrated only when the initial test of recollection included specific questions that later reappeared on the final recollection test [42]. This suggests that it might be of importance whether the immediate recall test examines the same details as the final test of recollection, or not.

The centrality of the details examined has been shown to matter: it is easier to create a misinformation effect for peripheral details compared to more central aspects and details of an event [43–46].

The time passage between the three steps typically used in misinformation studies is found to be of importance: a misinformation effect is favored by a long interval before the introduction of the misleading information, and by a short interval following it [20, 47]. This is in consonance with the discrepancy detection principle, which states that the likelihood of a person’s recollection being affected by the misinformation is larger if the person does not detect the discrepancy between the misinformation and the original event [48]. This principle applies to choice blindness because choice blind participants per definition do not detect the discrepancy between the misinformation and their recollection of the original event.

The current study follows the experimental steps of the misinformation effect paradigm and aims to, facilitated by choice blindness, make the participants seemingly present themselves with misleading information. The purpose of the current study was thus to first examine whether choice blindness can be created for recollections of a witnessed event. Based on previous choice blindness studies we hypothesized that it could. A further purpose was then to examine whether choice blindness affects later recollection of the event, in line with the misinformation effect. Previous studies of the source of the misinformation have indicated that a greater misinformation effect can be created if the source is rendered reliable, which can be achieved when the source is unbiased or has high-credibility or knowledge about the event. In the current study we investigate the source of the misinformation—who can you really rely on? On a daily basis you have to rely on your own mind to provide you with correct information about the world around you—you have to trust yourself to be reliable. What then if you were your own source of misleading information? Most likely, you trust yourself even when you are wrong. This was put to test, as the participants ostensibly supplied themselves with misinformation when their original recollections were manipulated and then presented to them as their own. To illustrate, they had to justify why they had said kidnapper in witnessed crime wore glasses, when in actual fact they had said he did not. As the source in the current study could be considered reliable we expected that a substantial misinformation effect would be created. We were also interested in how other variables would affect the creation of a misinformation effect i.e. the influence of an immediate recollection test; the centrality of the details examined; and the time interval between the different steps. Based on former studies it is difficult to hypothesize about the direction of impact of an immediate recollection test, as the results have been conflicting. In line with earlier research we speculated that the central details we examined would influence a misinformation effect negatively. According to previous research a short time interval between the witnessing of an event and the introduction of misinformation leads to a smaller misinformation effect. According to the discrepancy detection principle, detection of the false post-event information leads to a smaller chance for a misinformation effect. We speculated, though, that choice blindness could mediate this, as choice blindness per definition ensures no detection of the misinformation. We reasoned that we could therefore achieve a considerable misinformation effect despite a short interval.

The purpose of the current study was hence to converge the misinformation effect and choice blindness paradigms and thereby investigate whether people can seemingly be their own source of misleading information, thus creating memory blindness.

## Methods

### Participants

Fifty-four volunteers (34 female) participated in the current study. Ages ranged from 19 to 63 years (M = 26.2, SD = 7.9). The study was marketed as an eyewitness experiment and the participants were mainly students recruited from different departments at Lund University. All participants gave written informed consent and also agreed to have the interaction audio recorded.

### Ethics statement

The study was approved by the ethical committee of the Department of Psychology of Lund University and has been conducted according to the principles of the Declaration of Helsinki.

### Procedure and materials

In short, the procedure of the experiment contained five steps: first the participants were shown a film of a kidnapping, and then they filled out a questionnaire about events in the film. After this their answers were manipulated using sleight-of-hand. A discussion about their manipulated answers followed in an attempt to create choice blindness. Finally, a second questionnaire was administered in order to test the effect of the potential choice blindness. A more detailed account follows.

The participants were tested singly and the experiments started off by showing each participant a four-minute film on a 42” screen. The film was shot from a first-person perspective and shows the kidnapping of a young woman from a bus stop. Two men disembark from a car and approach the woman, then make her unconscious using chloroform. In the commotion the older, glasses-wearing man pulls out a gun, whilst the younger, cap-wearing man drags the woman into their car before they drive off from the scene. The film has been used in previous eyewitness research [49–51].

After the film was shown, the participants filled out a two-paged *statement questionnaire*, containing 20 statements about the film (e.g. The older man wears glasses). The questionnaire was influenced by the design used by Hall et al. [4]. Six of the 20 statements were target items (Table 1) that were manipulated, discussed with all participants, and the basis of the data analysis. The remaining 14 were filler items. For half of the participants items 9, 15 and 20 (version one) were manipulated, and for the other half items 6, 12 and 17 (version two) were manipulated. Every other participant was assigned to version one, and every other to version two. Thus, the different versions functioned as controls to one another. (See S1 and S2 Files for details)

**Table 1:** Statements and their corresponding questions in the two questionnaires.

Version 1		Version 2	
Statements	Corresponding questions	Statements	Corresponding questions
The older man jumps into the car and drives away^a^	Who drives the car?	A yellow car stops at the bus shelter^a^	What is the color of the men’s car?
The younger man holds a knife to the woman^a^	Does the younger man have a knife?	The younger man hits the woman in the head^a^	Does the younger man hit the woman in the head?
The younger man wears a cap^b^	Does the younger man wear a cap?	The older man wears glasses^b^	Does the older man wear glasses?

^a^The correct answer to the statements is “disagree.”

^b^The correct answer is “agree.”

The participants were instructed to answer each statement by marking a 96 mm long continuous scale, where a mark made to the left signified “Disagree”, a mark made to the right signified “Agree”, and a mark made somewhere in the middle of the scale signified “Uncertain.” If the participants had no recollection of a particular statement whatsoever, the mark was thus made on the absolute midpoint of the scale. Several previous choice blindness studies have also used continuous scales for ratings [3, 4, 14, 18, 52].

**Fig 1:**
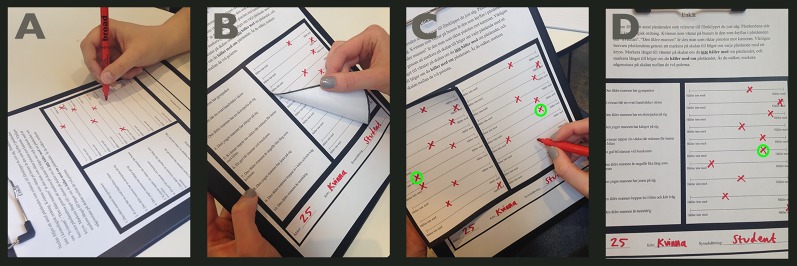
A staged demonstration of how the manipulations were carried out. (A) The participant fills out the statement questionnaire, by indicating level of agreement on a scale. (B) Experimenter A pulls away the sticker with the participant’s answers. (C) The experimenter fills out a new scale, which was hidden underneath the sticker, and manipulates some of the answers. (D) The new, manipulated questionnaire.

After the participants completed filling out the first sheet of the statement questionnaire, they handed it over to experimenter A, and continued with the second sheet of the questionnaire. Whilst the participants were busy filling out the second sheet, experimenter A manipulated one statement answer on the first sheet. In order to perform the manipulations the questionnaires were cleverly constructed using reattachable stickers (Fig 1). Meanwhile, experimenter B timed the filling out of the two sheets.

There was no predetermined rule for the size of the manipulations across the scale. Instead, the manipulations were made so that the markings were to signify a different answer along the scale, i.e. “Agree”, “Disagree” and “Uncertain” as described above, than the answer originally given by the participants, therefore making some of the manipulations sizeable. Also, each manipulation was made with the intent of creating a believable pattern of responses, mimicking the response pattern of each participant. For example, if a participant frequently marked towards the poles of the scale, the manipulations used this same pattern. Manipulations ranged from 32.0 mm to 96.0 mm and the mean distance of the manipulations was 55.7 mm (SD = 17.5) on the 96.0 mm scale. Incorrect answers were also manipulated along the scale, in the direction of the correct answer.

While the second sheet was also manipulated in the same manner as the first, the participants were busy chatting with experimenter B. All in all three statement answers were manipulated.

Experimenter B then discussed the same ten predetermined statements with all participants. For every predetermined statement, the participants were asked to describe their recollection of what they had seen on the film, and justify this. During the discussion notes were kept by experimenter A, and a sound recording was also made to support these.

After this discussion, the participants were given *the memory questionnaire* to fill out. The purpose of the memory questionnaire was to examine whether the manipulations made in the statement questionnaire would affect the participants’ recollection of what they had witnessed on the film. It consisted of 15 questions, six of which were related to the six target items of the statement questionnaire. This questionnaire had no scale. For eight of the questions the participants chose between the alternatives “Yes”, “No” and “Don’t know.” In several studies of the misinformation effect [19, 26] a forced choice design is used in the memory questionnaire, where the participants must choose either”Yes” or”No” for the question probing the misinformation given earlier. This is a poor reflection on reality, where people are seldom forced to give their recollection of an event if they do not have one. Therefore, we chose to give our participants the chance to say that they did not know. The remaining seven questions were answered in free response, where the participants also had the choice to say they did not know.

The two questionnaires hence had different designs. There was no credible reason for the participants to fill out a questionnaire identical to the one they had just filled out and discussed a few minutes ago. There was also a risk that the participants would strive to give the same answer in both questionnaires, rather than try to remember what they had witnessed on the film. The scale in the statement questionnaire was therefore not graded in any way, thus making it unlikely for the participants to think of their answers in the dichotomous yes-or-no-terms used in the memory questionnaire. To further curb this consistency endeavor, the participants were also explicitly asked to give their recollection of the film for the final questionnaire.

To facilitate a comparison between the two questionnaires, and thereby examining if a misinformation effect had occurred, the answers in the statement questionnaire were categorized. The participants were not aware of this categorization when they gave their original answers. To give them a fair chance to detect that the manipulation did not reflect their original opinion, the categories were verbalized during the discussion by the experimenter, for example”The older man wears glasses… you *disagree with that*.”

Finally, in order to find out whether they had suspected that any of their answers on the statement questionnaire had been manipulated experimenter A asked the participants, in accordance with other choice blindness studies [2, 4, 10, 13, 18], four detection questions: “How did this feel?” “Did you think of anything in particular during the experiment?” “Did you think of anything in particular when the two of you discussed the statements?” and “Did you notice anything strange about the questionnaires?” After this the true purpose of the study was revealed. During the experiment there was no delay between the different steps, and no filler task was used.

### Measures

The participants were categorized as either non-detectors or detectors. Non-detectors were those who at no point during the experiment expressed any kind of suspicion concerning the manipulations. These participants, however, could still correct the manipulated answers, but without suspecting that the answers were not their own. The inclusion of these participants in the subsequent analysis is in accordance with previous choice blindness studies [3, 4, 11, 12, 52, 53]. The participants expressed that they had misread the statement or that they had marked the wrong end of the scale: “I was sloppy when I filled this out. I’m sorry! I’m pretty sure he doesn’t hit her in the head.” These participants were then asked if they wanted to correct their marking to better match their recollection.

Detectors, however, expressed suspicion concerning the manipulations. *Concurrent detectors* clearly stated that the manipulated answers were not their own during the discussion about the statements. *Retrospective detectors* were those who, during the four detection questions at the end of the experiment, expressed that their answers had been manipulated in some way. Both concurrent detectors and retrospective detectors were removed from the subsequent analyses.

In order to quantify the size of the choice blindness manipulations across the scale, the difference between the markings originally made by the participants and the manipulated markings was measured in millimeters. To then examine whether the answers in the memory questionnaire were affected by the manipulations made in the statement questionnaire, the scale was divided into three equally sized parts. A marking made in the part to the left was categorized as “No”, a marking made in the middle part was categorized as “Don’t know”, and a marking made in the part to the right was categorized as “Yes.”

These three categories were chosen because these were the three response alternatives in the memory questionnaire, thus allowing a comparison between the two questionnaires. The answers in the memory questionnaire were categorized in the following way: “Changed in the direction of the manipulation,” “Changed independently of a possible manipulation” and “Not changed.”

The first category “Changed in the direction of the manipulation” meant that a choice blind participant’s answer in the memory questionnaire did not belong to the same category as that given in the statement questionnaire. For example, if the participant’s answer in the statement questionnaire was manipulated from “Disagree” to “Agree”, and the participant later gave the answer “Yes” or “Don’t know” in the memory questionnaire, the recollection would have been changed in accordance with the manipulation, as the expected answer would be “No.” The second category “Changed independently of a possible manipulation” also meant that the participant’s answer in the memory questionnaire did not belong to the same category as that given in the statement questionnaire. However, this category was used either because a choice blind participant had changed their answer in the opposite direction of the manipulation, or because a participant whose markings had not been manipulated answered differently in the two questionnaires. The third and final category “Not changed” meant that the same answers were given in both questionnaires.

In the main statistical analysis of the transference between the two questionnaires, item 6, “A yellow car stops at the bus shelter” and item 9, “The older man jumps into the car and drives away” were discarded, in part because they were answered in free response in the memory questionnaire, and in part because these items were somewhat ambiguous in their nature. Categorizing the free response answers involved a great deal of interpretation, which was deemed too arbitrary. The ambiguity in the wording of the statements made the participants likely to reinterpret them as they justified markings they themselves had not made. After a reinterpretation of the statement, this no longer matched the corresponding question in the memory questionnaire. Including the two ambiguous items, however, did not change the overall misinformation effect outcome. That is, if all six items were included in the analysis a similar result, but with a somewhat smaller effect size, was still evident when comparing recollection of choice blind items with non-manipulated items, χ2 (2, n = 196) = 92.4, p < .001, Cramer’s *V* = 0.69, p < .001.

## Results

### Choice blindness

Three participants were categorized as concurrent detectors, and an additional five as retrospective detectors. These participants were removed from the subsequent analyses. The remaining 46 participants were categorized as non-detectors. These were either choice blind or, when they corrected the statements, expressed that they had misread the statement or that they had marked the wrong end of the scale, and had no suspicion of any manipulations.

Each participant had three answers manipulated. The mean distance of the manipulations was 55.7 mm (SD = 17.5) on the 96.0 mm scale. Of the three manipulated answers, each participant corrected a mean of 1.80 (SD = 0.92) answers, meaning that each participant was choice blind for 1.20 (SD = 0.92) of the manipulated answers. There was no significant difference on the number of corrected items between version one (M = 16.7, SD = 9.2) and version two (M = 19.3, SD = 9.2; *t* (52) = -1.04, p = .30) of the statement questionnaire. In total, 161 manipulations were made, and 61% of these were corrected, which leaves 39% of the manipulations for which choice blindness were created. On an individual level, 28% of the participants corrected all three manipulations, while 72% of the participants were choice blind for at least one of the three manipulations.

In the correction process for the manipulations during the discussion, the participants were allowed to change their marking to better match their recollection. The corrected markings the participants made (M = 1.6, SD = 1.7) differed significantly from their original ones (M = 1.1, SD = 1.5; *t* (79) = 3.3, p = .001). 64% of these corrections were made in the direction of the manipulation, 19% were made in the opposite direction of the manipulation, and 17% exactly matched the participants’ original markings. Participants moved their markings on average 8.18 mm in this correction process.

The number of corrected answers was not significantly related to gender or age, nor was it related to the time the participants took to fill out the statement questionnaire (see S1 Table for details). The distance of the manipulations across the scale differed between corrected answers (M = 59.9, SD = 18.6) and non-corrected answers (M = 49.3, SD = 13.3; *t* (158) = - 3.86, p < .001). A correction on the first of the three manipulations did not create a larger amount of correction for the second manipulation, χ2 (1, n = 53) = 0.40, p = .53, phi = 0.13. Such a cascading effect was also absent for correction on the first or second manipulation on the third manipulation, χ2 (1, n = 54) < .001, p = 1.0, phi > 1.00. The correctness of the answers in the statement questionnaire was not significantly linked to the occurrence of choice blindness, χ2 (2, n = 161) = 4.93, p = .09, Cramer’s *V* = 0.18. Participants gave the incorrect answer in 18% of the statements where choice blindness occurred.

Two of the manipulated statements contained elements of ambiguity, namely”A yellow car stops at the bus shelter” and”The older man jumps into the car and drives away.” When choice blindness was created for these two statements, the participants typically justified the false choice by reinterpreting the statement, e.g.”I just mean that he jumps in, but he’s not the one driving.” This is an example of a participant trying to justify an answer in the middle of the scale for the statement about the older man driving the car, when they had not agreed originally. In the memory questionnaire, the corresponding question to this statement was “Who drives the car?” However, as the statement is reinterpreted, this corresponding question no longer becomes relevant as a mean of examining a change in recollection, hence these items were, as described above, excluded from the following main analysis of transference between the two questionnaires.

### Misinformation effect

In the following, our main focus is the comparison between the non-manipulated statements and the manipulated statements where choice blindness occurred. That is, we are not mainly interested in the corrected manipulated statements where choice blindness did not occur.

**Fig 2:**
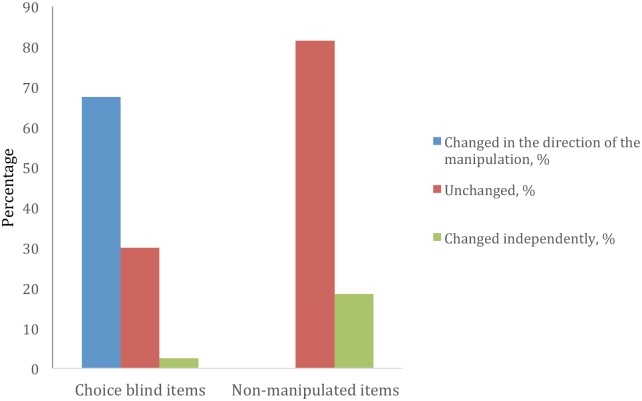
The answer consistency between the statement questionnaire and the memory questionnaire.

In 70% of the statements where choice blindness occurred, a different answer was reported in the memory questionnaire compared to the statement questionnaire. 68% of the answers were changed in the direction of the manipulations and 2% were changed independently of the manipulations. In 19% of the non-manipulated statements, a different answer was reported in the memory questionnaire compared to the statement questionnaire (Fig 2). Thus, there was a significant difference between statements for which choice blindness occurred and non-manipulated statements regarding the consistency of answers given in the statement questionnaire and answers given in the memory questionnaire, χ2 (2, n = 132) = 78.6, p < .001, with a large effect size, Cramer’s *V* = 0.77, p < .001. The participants thus changed their answers to a significantly larger degree in statements where choice blindness was created.

When comparing the manipulated statements where choice blindness occurred with the manipulated statements where choice blindness did not occur, the same difference in answer consistency can be seen, χ2 (2, n = 92) = 33.4, p < .001, with a large effect size, Cramer’s *V* = 0.60, p < .001. That is, also in this comparison the participants changed their answers to a significantly larger degree in statements where choice blindness occurred.

## Discussion

The absurd way in which Orwell’s citizens of Oceania allow their memories to be manipulated seems extreme. However, this study indicates that in some situations, with relatively simple means, one can convince people to accept and endorse a recollection of an event that they have not originally reported, and that this can make them believe that they always recalled the event in this manner. The results show that choice blindness can be created for people’s recollections of a witnessed event, and these false recollections can in turn create a change in the reported remembrance of the witnessed event.

The misinformation effect has been found to be highly dependent on who delivers the misleading information, i.e. who the source is [26–31]. Previous studies have used a number of different sources: a lawyer, the culprit [26], a co-witness [23–25], or a professor [25]. As first shown by Dodd et al. [26] a larger misinformation effect is created when the participants find the source reliable and trustworthy. By accepting the manipulations as their own answers, and then justifying why they answered the way they did(n’t), the participants in the current study in effect acted as *their own source of misleading information*. The vividness of the false recollection was entirely dependent on the participants’ own introspections, and creating a detailed image of the false information may have strengthened their recollection of it. These results indicate that perhaps we trust no one better than we trust ourselves.

In the current study, a large majority of the participants were choice blind for at least one of the three manipulations, and choice blindness was created for almost half of the manipulations. These effect sizes are larger than those obtained in the field study of eyewitnesses’ facial recognition [13]. Their study only manipulated one item per participant, and the number of choice blind participants was 41%, while in the current study the corresponding percentage is 72%. This is surprising because the conditions in Sagana et al. are favorable for choice blindness, i.e. a field study and a non-crime-situation. In contrast our study used an experimental design and a forensic setting where participants are highly motivated to accurately remember the criminal event. Furthermore, in the Sagana et al. [10] follow up study, they too used films in a crime-situation, but found very limited, or no, choice blindness.

A questionnaire akin to the one used in the current study was used by Sauerland et al. [18] to examine choice blindness for participants’ own history of norm violating behavior. As opposed to our design, where the questionnaires never left the participant’s field of vision, the experimenter left the room with the filled out questionnaire for ten minutes. This probably made it more likely for the participants that something could have happened to their answers, which could have contributed to the relatively low rate of choice blindness in their study (i.e., 10%). An additional difference between their study and ours is that they examined choice blindness for autobiographical memory, and we examined choice blindness in relation to episodic memory.

We found no significant connection between correctness of the participants’ original answers and whether they were subsequently choice blind, and neither did Sagana et al. [13]. Correctness can therefore probably be excluded as a confounding variable.

However, a significant relation was found between choice blindness and the distance the original answers had been manipulated across the scale. Corrected answers had a larger mean manipulation distance than non-corrected answers. Hall et al. [4] found no significant relation between manipulation distance and choice blindness. One explanation to this discrepancy could be that the mean manipulation distance in our study was greater than theirs. Our larger manipulations were motivated by an attempt to move the participants’ answers far enough across the scale to represent a different answer than they had originally intended.

The false recollections justified by the choice blind participants created a change in their reported remembrance of details from the witnessed event, something none of the previous studies combining choice blindness and memory have achieved. A large majority of the choice blind answers were changed in the direction of the manipulations between the statement questionnaire and the memory questionnaire. Only a small minority of the non-manipulated answers was changed between the two questionnaires. This indicates the creation of a strong misinformation effect.

Cochran et al. [14] could in their first experiment, on episodic details, not present a clear number of detectors and non-detectors of their manipulations. They could thus not conclude whether it was choice blindness, or the mere exposure to the manipulations, that affected the subsequent change in recollection of details from the witnessed event between the two testings. The results from the current study could help fill this blank, as our study clearly shows that only the choice blind items have a misinformation effect. This consequential effect is accordant with previous choice blindness studies using repeated measures [10, 11] and also with the discrepancy detection principle [48], and thus shows that the phenomenon can be generalized to a memory setting as well. It is therefore meaningful to employ the term memory blindness, which was first proposed by Cochran et al. [14] for this phenomenon. We interpret the memory blindness term to mean the failure to detect a discrepancy between a reported recollection of a witnessed event and a falsified recollection, and to then adopt this false recollection as a memory.

The fact that the Cochran et al. [14] study was conducted online provides additional arguments that this proposed phenomenon is not an artifact due to unwillingness to report inconsistencies to an experimenter. The advantages of a lab setting are that the participants are likely to be attentive, perform their best and pay attention to the instructions given. It gives the opportunity to clarify conceivable misunderstandings and also makes for a convincing choice blindness manipulation. Conversely, there are also certain limitations with such a design, which in an online setting might be mitigated; participants might refrain from reporting detection in person and they might feel obliged to appear consistent to the experimenters, and thus in the final questionnaire hold on to the answers they had just previously given justifications for. Such a motivation is further challenged by the fact that all but three participants in the current study were willing to, during the discussion with the experimenter, be inconsistent with answers they had supposedly given just moments earlier in the statement questionnaire, as they chose to make corrections of these.

In order to make the choice blindness element of the design somewhat more stringent, future research could further increase the power of the four detection questions asked in the end of the experiment by forthright claiming that for some of the participants their responses have been altered before given back to them and then put the question “Did you notice whether any of your answers were changed?” If they respond positively to this they would then indicate which of their answers they believe have been altered. If instead giving a negative response this would then suggest choice blindness blindness [2, 10].

The current experiment follows the three basic steps of the misinformation effect paradigm: the witnessing of an event, the receiving of misleading information and the test of recollection [19]. There is, however, also an additional step, namely an immediate recollection test—the statement questionnaire. Previous studies have shown conflicting results regarding the direction of influence of such a test. The results of the current study seem to suggest that an immediate test of recollection after witnessing an event is not an immunizer to a misinformation effect as indicated by Gabbert et al. [36], Memon et al. [37] and Wang et al. [38]. The statement questionnaire and the memory questionnaire used in the current study examine recollections of the same details, and the results are in line with the findings by Wilford et al. [42]. These suggested that a retrieval enhanced suggestibility effect was only demonstrated when the two tests examined the same details. The use of consecutive tests also, however, presents the current study with certain limitations. That is, the answers in the final memory questionnaire might reflect certain response biases following the initial statement questionnaire and the subsequent discussion, thus making exhaustive inferences from the current results difficult, in terms of a long-lasting memory change.

The current study examines only details central to the event—aspects that would be of importance if the participants really had witnessed the kidnapping in real life. The participants were likely to attend to these details, and in spite of this a misinformation effect occurred. This opposed to what previous studies have suggested, namely that it is easier to create a misinformation effect for peripheral details compared to more central aspects and details of an event [43–46].

The misinformation effect is larger the shorter the time interval between the introduction of the misleading information and the subsequent memory test [20, 47]. In the current study, there was no interval between introducing the misinformation and the testing of memory, and this could be a contributing factor to the relatively large misinformation effect that occurred. However, the misinformation effect also benefits from a long interval between the witnessed event and the later introduction of misleading information [20, 47]. In the current study there was virtually no time interval between witnessing and misinformation, but yet a considerable misinformation effect occurred. A short time between these two steps could increase the risk of detecting the misleading information. However, as stated by the discrepancy detection principle, if not detected, there is a great chance for a misinformation effect, perhaps made bigger by the short time interval. The exceptional factor in the current study is the choice blindness element, which makes it possible to, with no delay, present the misinformation without detection. Choice blindness thus functions as a catalyst for a misinformation effect, and combined they create memory blindness.

One limitation to the present experiment is the difficulty making inferences regarding conceivable long lasting memory changes as a consequence of the displayed choice blindness. This difficulty making inferences concerning the factual impact of post-event information has been the topic of much debate [54]. One could argue that the reporting of misinformation could indicate a response bias in line with the misinformation, rather than an underlying memory change. As previously mentioned potential response biases may be hard to rule out, however there might be ways for future studies to counter this. One way to bypass this could be to post-warn participants about earlier misinformation, which could reduce a misinformation effect [55]. This is true also after a misinformation effect has been obtained [56]. However, we argue that the fact that almost all participants in the current experiment “discovered”, or corrected, at least one of the three manipulations encouraged scrutiny, and hence discouraged relying too much on the previously given account. Nevertheless, it would have been possible to introduce post-warnings in the current experiment by asking the four questions regarding detection of the manipulations before the memory questionnaire, where the subsequent memory answers could signify a more robust, lasting change in recollection. The detection questions would thus function as a post-warning of the misinformation and might encourage a more sensitive source monitoring. This would have provided stronger evidence of a lasting misinformation effect and memory change, rather than a changed report of recollection, which might derive from potential response biases. The design of the current study, however, is more consistent with real life, where warnings typically are not provided, nor are people frequently made wary of possible inconsistencies in their testimony.

We conclude, that the choice blindness, presumably due to its possibilities of without awareness replacing the outcome of a choice, may have given the misinformation effect extra strength, where the key factor is that the misinformation originates from the participants themselves.

## Conclusions

The current study was designed to, in a live setting, create choice blindness for participants’ recollections of a witnessed event and thus also create changes in their recollection, a new approach to the misinformation effect literature. The results are clear—people can, and are even *likely* to, be blind for their own recollections of an event. This can be achieved to the extent that they can justify something that never happened, and something they themselves initially never thought had happened, as was done by a majority of the participants in the current study. A majority of the choice blind participants were affected by the misleading information as indicated by their low answer consistency between the statement and memory questionnaires, compared to the considerably higher consistency for the non-manipulated items. Memory blindness was thus created.

The noteworthy factor in this study was the way in which the participants seemingly delivered the faulty information to themselves, and consequently affected the way they later reported the witnessed event. This was done with no delay, no filler task, and no distractions, and the manipulations were carried out virtually right under the participants’ noses.

## Supporting information

S1 File The statement questionnaire. (DOCX)

S2 File The memory questionnaire. (DOCX)

S1 Table Non-significant tests reported in results section. (DOCX)
