# Supplementary material for: Self-delivered misinformation - Merging the choice blindness and misinformation effect paradigms
Source: PLoS One. 2017 Mar 8;12(3):e0173606. doi: 10.1371/journal.pone.0173606 (PMC5342302; doi:10.1371/journal.pone.0173606)
Supplement: S1 File — (DOCX) [file pone.0173606.s001.docx]

**S1 File. The Statement Questionnaire.**

Below follows a number of statements related to the film clip you just watched. The statements are not written in a chronological order. It is the woman waiting for the bus who is intended in the statements concerning “the woman”. ”The older man” is the man who aims his gun towards the camera. Please answer the statements by putting a mark on the scale to the right of each statement. Put a mark leftmost if you disagree with the statement, and rightmost if you agree with the statement. If you are uncertain, put a mark somewhere along the scale between the two extremes.

1. The older man wears sneakers
2. The woman carries a black leather purse
3. The older man wears a leather jacket
4. The younger man wears boots
5. The woman drops her purse when the men lead her to the car
6. A yellow car stops at the bus shelter
7. The older man is approximately the same height as the woman
8. The younger man wears jeans
9. The older man jumps into the car and drives away
10. The older man is balding
11. The woman wears a black-and-white maxi dress
12. The younger man hits the woman in the head
13. An older gentleman and with a dog observes the incident from across the street
14. The men approach the woman and the older man shows her something
15. The older man wears glasses
16. The car that stops at the bus shelter has license number PAA 006
17. The younger man holds a knife to the woman
18. Time is a quarter past one
19. The woman wears a black jacket
20. The younger man wears a cap

*Note*. The instructions and statements from the statement questionnaire translated from Swedish.
