# Supplementary material for: Self-delivered misinformation - Merging the choice blindness and misinformation effect paradigms
Source: PLoS One. 2017 Mar 8;12(3):e0173606. doi: 10.1371/journal.pone.0173606 (PMC5342302; doi:10.1371/journal.pone.0173606)
Supplement: S2 File — (DOCX) [file pone.0173606.s002.docx]

**S2 File. The Memory Questionnaire.**

Below follows a number of questions about the film clip you just watched. Please answer the questions in the area provided to the right. For questions with a yes-or-no answer, please choose one of these. For the remaining questions give your answer in free response.

1. Does the older man wear glasses?
2. What is the license number of the men’s car?
3. Who drives the car?
4. Does the younger man wear a light color shirt?
5. Does the younger man hit the woman in the head?
6. Does the woman wear a patterned maxi dress?
7. How many people are involved in the kidnapping of the woman?
8. What is the color of the woman’s jacket?
9. What time is it?
10. Does the younger man have a knife?
11. Does the woman wear her hair in a hair clip?
12. Does the younger man wear a cap?
13. What is the color of the men’s car?
14. Is the woman drugged?
15. What is the color of the woman’s hair?

*Note*. The instructions and questions from the memory questionnaire translated from Swedish.
