# Supplementary material for: Self-delivered misinformation - Merging the choice blindness and misinformation effect paradigms
Source: PLoS One. 2017 Mar 8;12(3):e0173606. doi: 10.1371/journal.pone.0173606 (PMC5342302; doi:10.1371/journal.pone.0173606)
Supplement: S1 Table — (DOCX) [file pone.0173606.s003.docx]

**S1 Table.** Non-significant tests.

| Variables compared | Statistical test | Descriptive + Test result |
| --- | --- | --- |
| Choice blindness by participant gender | Chi-squared test for  independence | χ2 (2, n = 54) = 3.21, p = .20 |
| Choice blindness by participant age | Logistic regression analysis | \| **Coefficient** \| **β** \| **S.E.** \| **Wald’s chi^2^** \| **df** \| **Sig. (p)** \| **Exp β** \| \| --- \| --- \| --- \| --- \| --- \| --- \| --- \| \| Constant \| .28 \| 1.35 \| .04 \| 1 \| .84 \| 1.32 \| \| Age \| -.05 \| .05 \| .85 \| 1 \| .36 \| .95 \| |
| Choice blindness by participants’ time to fill out the statement questionnaire | Logistic regression analysis | \| **Coefficient** \| **β** \| **S.E.** \| **Wald’s chi^2^** \| **df** \| **Sig. (p)** \| **Exp β** \| \| --- \| --- \| --- \| --- \| --- \| --- \| --- \| \| Constant \| -3.16 \| 1.28 \| 6.11 \| 1 \| .01 \| .04 \| \| Time \| .01 \| .01 \| 3.31 \| 1 \| .07 \| 1.01 \| |
